# Distal and local mucosal immunization with a BoHV-4-based vector delivering CpHV-1 gD confers protection against intravaginal CpHV-1 challenge in goats

**DOI:** 10.3389/fimmu.2026.1884557

**Published:** 2026-07-14

**Authors:** Amienwanlen Eugene Odigie, Sergio Minesso, Valentina Franceschi, Grazia Greco, Vittorio Madia, Michele Camero, Maria Tempesta, Gaetano Donofrio

**Affiliations:** 1 Department of Veterinary Medicine, University of Bari, Valenzano, Italy; 2 Department of Veterinary Science, University of Parma, Parma, Italy

**Keywords:** BoHV-4 vector, caprine herpesvirus 1 (CpHV-1), glycoprotein D (gD), intravaginal and intranasal immunization, mucosal vaccination, protective immunity

## Abstract

Effective vaccines against sexually transmitted pathogens must elicit protective immunity at mucosal surfaces, particularly within the female reproductive tract. Caprine herpesvirus 1 (CpHV-1) causes genital disease, reproductive failure, and neonatal mortality in goats and provides a biologically relevant large-animal model for human genital herpesvirus infection. Here, we evaluated a mucosal vaccination strategy based on a bovine herpesvirus 4 (BoHV-4) vector expressing the CpHV-1 immunodominant glycoprotein D (BoHV4-A-gD(cp)gD(106)ΔTK). Goats were immunized via the intravaginal or intranasal route using a prime–boost regimen and subsequently challenged intravaginally with virulent CpHV-1. *Ex vivo* tissue analyses showed efficient transduction of nasal mucosa and limited vaginal transduction restricted to the cervical region. Despite these differences, both immunization routes conferred complete protection against disease. Vaccinated animals showed no fever or genital pathology following challenge, in contrast to unvaccinated controls. Viral shedding was significantly reduced in vaccinated goats, with intravaginal immunization providing superior control of genital virus excretion. Both routes induced CpHV-1–specific systemic antibody responses, including functional neutralizing antibodies, with higher neutralizing titers observed after intranasal vaccination. These findings demonstrate that BoHV-4–based vectors can induce robust protective immunity against genital herpesvirus infection when delivered via either local or distal mucosal routes. The results highlight the importance of mucosal vaccination strategies and support the versatility of BoHV-4 as a vaccine platform for sexually transmitted infections. This study further establishes the goat–CpHV-1 model as a valuable translational system for the preclinical evaluation of mucosal herpesvirus vaccines.

## 1 Introduction

According to WHO estimates, approximately one million sexually transmitted infections are acquired every day, predominantly by women living in marginalized regions of low-income countries [https://www.who.int/news-room/fact-sheets/detail/sexually-transmitted-infections-(stis)]. These infections are not only associated with severe reproductive complications but can also lead to death. Although effective treatments exist, logistical and economic barriers, limited access to healthcare infrastructure, and persistent social stigma often prevent timely intervention, thereby worsening disease outcomes. For these reasons, preventive approaches may represent a more effective strategy to overcome the limitations associated with therapy.

Vaccination remains the most powerful tool for preventing transmissible diseases. For example, human papillomavirus (HPV) vaccination campaigns have saved thousands of lives by preventing cervical cancer (1). The vagina represents a major entry point for sexually transmitted pathogens, and the vaginal mucosal immune system constitutes the first line of defense. Consequently, intravaginal vaccination may offer a promising strategy to induce protective immunity directly within the genital tract. Exposure of the vaginal mucosa to specific pathogen antigens can first elicit innate immunity and subsequently trigger adaptive responses, ultimately establishing immune memory in regional lymph nodes and enabling faster and more efficient pathogen clearance (1, 2).

Caprine herpesvirus 1 (CpHV-1, now renamed Varicellovirus caprinealpha1) is a member of the order *Herpesvirales*, family Orthoh*erpesviridae*, subfamily *Alphaherpesvirinae*, and genus *Varicellovirus* (3, 4). In goats, CpHV-1 causes two main clinical forms: a fatal systemic infection in kids (5) and a genital disease in adults, manifested as vulvovaginitis and abortion in females (6, 7) and balanoposthitis in males (8). CpHV-1 infection begins at the respiratory or genital mucosa and then spreads systemically via a mononuclear cell–associated viremia, causing abortion in pregnant goats (7, 9). Viral shedding occurs through ocular, nasal, and genital secretions, with the genital tract representing the main site of viral entry and herd persistence (10). Abortions, usually occurring in mid-to-late gestation, can be experimentally induced by intranasal or intravenous inoculation (11). Following intravaginal infection, CpHV-1 establishes latency in the sacral ganglia, from which it may reactivate under physiological stress during the mating season, although experimental reactivation requires high-dose dexamethasone (12). CpHV-1 shares key biological features with HSV-2 (Human herpesvirus 2) and BoHV-1 (Bovine herpesvirus 1), including similar biological characteristics, vaginal epithelial tropism, genital lesions, and latency in the sacral ganglia (13, 14). These shared features position CpHV-1 as a unique dual-purpose model, serving as a large-animal model for investigating human genital herpesvirus infections and as a small-animal model for studying bovine genital disease.

A key prerequisite for successful vaccination is the choice of an effective antigen-delivery system, and viral vectors currently represent one of the most promising platforms (15–20). Bovine herpesvirus 4 (BoHV-4) is a gammaherpesvirus frequently detected in both healthy cattle and animals presenting with reproductive or respiratory disorders (21–23). Although growing evidence suggests that BoHV-4 may act as a secondary contributor to bovine postpartum metritis, its pathogenic role remains unclear (23–26). Despite its genomic classification within the Gammaherpesvirinae, BoHV-4 displays atypical biological features, including a pronounced cytopathic effect and broad replication capability in primary cells and cell lines from multiple species. Unlike other gamma herpesviruses, no oncogenic or transforming activity has been associated with this virus. Notably, BoHV-4 can incorporate large foreign DNA fragments without impairing replication, supporting its development as a versatile platform for gene delivery and oncolytic applications (27).

The safety and efficacy of the BoHV-4–based vector as a vaccine platform for protecting goats from CpHV-1–induced genital disease were previously demonstrated (28). In the present study, we evaluated whether mucosal immunization—either locally via the intravaginal route (IVa) or distally via the intranasal route (INa)—with a BoHV-4–based vector expressing the immunodominant glycoprotein D of CpHV-1 (BoHV-4-A-gD(cp)gD(106)ΔTK) (28) could protect goats from intravaginal challenge with highly pathogenic CpHV-1. Our results demonstrate that mucosal immunity induced either locally or distally provided complete protection against local infection and female reproductive tract pathology.

## 2 Results

### 2.1 Efficient nasal mucosal and cervical-restricted vaginal transduction by BoHV-4–based vector

**Figure 1:**
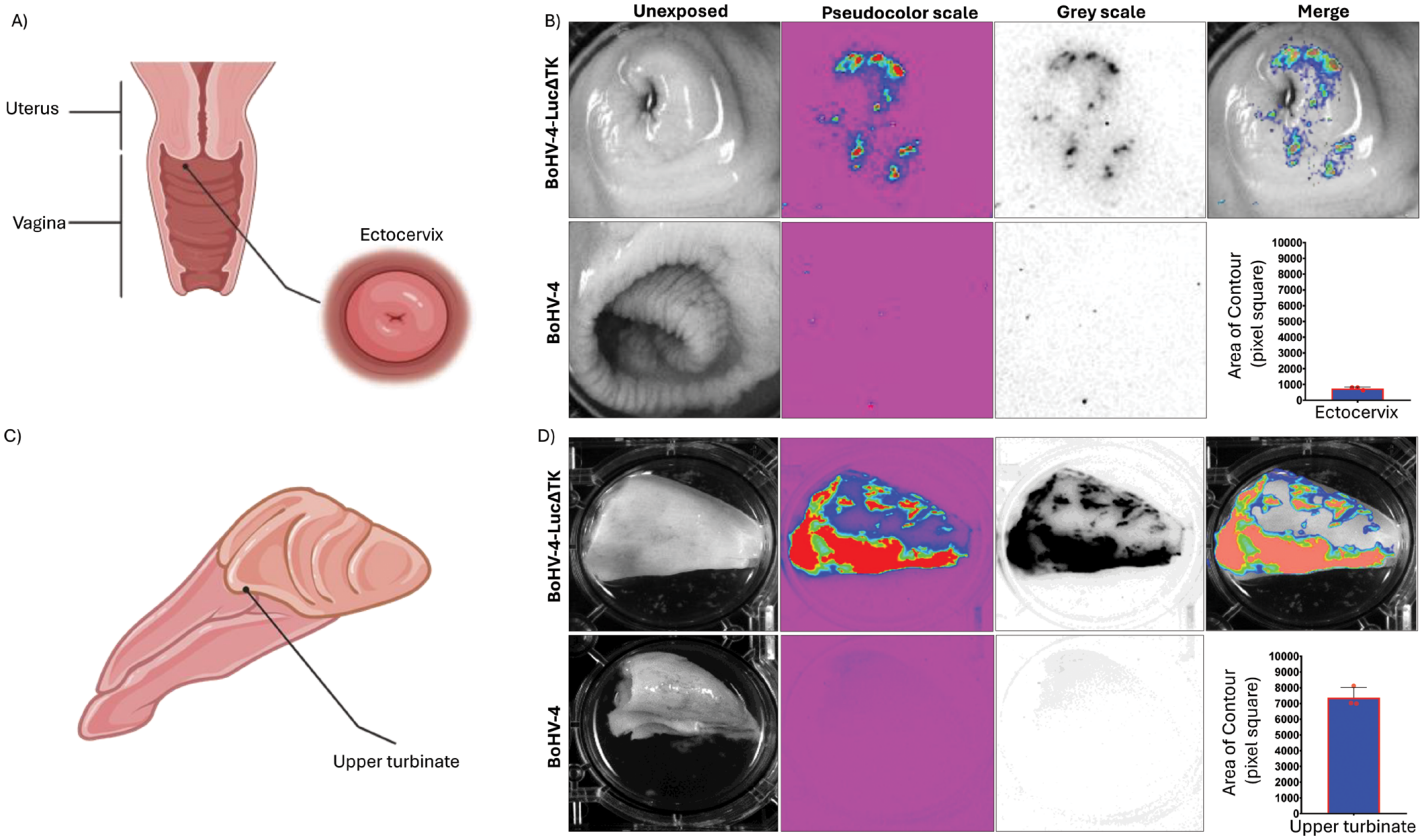
Ex vivo image analysis of BoHV−4−LucΔTK–transduced nasal turbinate and ectocervix. **(A)** Representative image showing a longitudinal section of the goat utero−vaginal tract together with a transverse section highlighting the ectocervix. **(B)** Representative bioluminescence imaging (BLI) of the ectocervix surrounded by the vaginal tissue following treatment with BoHV−4−LucΔTK or BoHV−4. Transduced tissues are shown in pseudocolor and grayscale, along with unexposed and merged images. Photon emission is quantified and expressed as pixel square. **(C)** Image illustrating the anatomical structure of the goat nasal turbinate and the localization of the upper turbinate used for treatment with BoHV−4−LucΔTK or BoHV−4. **(D)** Transduced tissues are shown in pseudocolor and grayscale, together with unexposed and merged images.

Before attempting the use of BoHV-4-A-gD(cp)gD(106)ΔTK for vaccination in goats via distal and local mucosal routes, we investigated the ability of this vector to transduce goat tissues *ex vivo*. A recombinant BoHV-4 expressing the luciferase enzyme under the control of the CMV promoter [BoHV-4-A-LucΔTK; (29)] was employed. Vaginal tissues, including the cervix, and nasal turbinate containing mucosa were collected at the abattoir from two slaughtered goats, dissected, and exposed to either BoHV-4-A-LucΔTK or wild-type BoHV-4 (negative control) in six-well tissue culture dishes. Twenty-four hours post-infection, samples were treated with a luminescent substrate and analyzed using *in vivo* imaging. As shown in **Figure 1A**, a strong luminescent signal was detected in all turbinate samples treated with BoHV-4-A-LucΔTK, whereas no signal was observed in samples treated with parental BoHV-4. In contrast, in vaginal samples, luminescence was detected exclusively in the cervical region and not in the pseudostratified epithelium of the vaginal mucosa (**Figure 1B**). These results demonstrate that the BoHV-4–based vector efficiently transduces the nasal mucosa, while vaginal transduction is restricted to the cervical area.

### 2.2 Mucosal immunization of goats with BoHV-4-A-gD(cp)gD(106)ΔTK confers clinical protection against CpHV-1 pathogenic challenge

**Figure 2:**
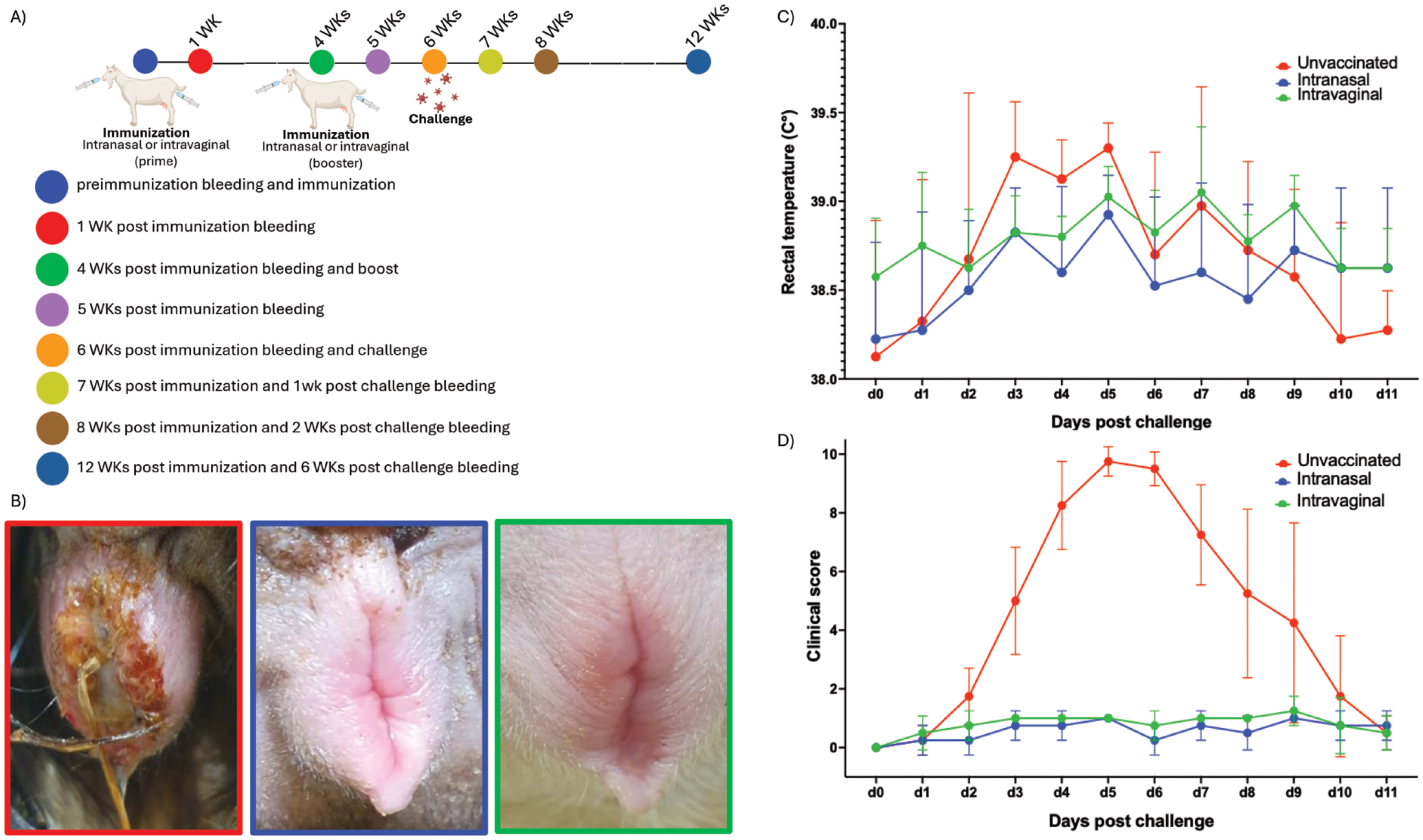
Clinical protection induced by distal and local mucosal vaccination with BoHV4-AgD(cp)gD(106)ΔTK. **(A)** Experimental immunization and CpHV-1 challenge timeline. Blood samples were collected before the first immunization (blue dot; prime), one week after the prime (red dot; 1 WK), at week 4 after the prime and prior to the booster (green dot; 4 WKs), 5 weeks after the prime (purple dot; 5 WKs), and 6 weeks after the prime and before intravaginal challenge with the BA-1 CpHV-1 strain (orange dot: 6 WKs). Additional samples were taken 7 (chartreuse dot; 7 WKs), 8 (brown dot; 8 WKs), and 12 weeks (sarin dot; 12 WKs) after the prime. **(B)** Representative images of vulvae following intravaginal CpHV-1 challenge: unvaccinated controls (red frame), INa group (blue frame), and IVa group (green frame). Control animals exhibit edema, vesiculo-ulcerative lesions, and fibrinous discharge, whereas vaccinated animals show no visible lesions. **(C)** Mean rectal temperature recordings of unvaccinated (red), INa (blue), and IVa (green) goats from day 0 (pre-challenge) to day 11 post-challenge. **(D)** Mean cumulative clinical scores of the three experimental groups over the same period indicating significant reduction of clinical manifestations in vaccinated groups. Data were analyzed with two-way ANOVA or Welch's ANOVA followed by pairwise Tukey HSD or Games-Howell posthoc comparison, respectively.

Although transduction of the vaginal mucosa by the BoHV-4–based vector was restricted to the cervical area, both the nasal and vaginal routes were selected for subsequent inoculation experiments. To evaluate the protective efficacy of the candidate BoHV-4-A-gD(cp)gD(106)ΔTK vaccine, an experiment was designed in which two groups of goats were immunized intravaginally (IVa, n = 4) or intranasally (INa, n=4) with the recombinant vector, while a third group served as unvaccinated controls (n=4) (**Figure 2A**). Vaccinated animals received a booster dose 28 days after the primary immunization. All goats were challenged on day 49 post-immunization with virulent CpHV-1. Blood samples were collected weekly from all animals throughout the study period (**Figure 2A**). Daily rectal temperatures and clinical signs were monitored in all experimental goat groups throughout the trial following challenge. Control goats developed mild pyrexia between days 3 and 7 post-challenge (**Figure 2B**), whereas vaccinated animals (BoHV-4-A-gD(cp)gD(106)ΔTK groups) maintained normal temperature values (**Figure 2C**). Overall, febrile responses did not differ significantly between the goats in the control and vaccinated groups (P>0.05) (**Table 1**).

**Table 1:** Result of Welch’s ANOVA analysis for virus excretion titre, IgG titer, neutralizing titer, temperature and clinical score.

Variable	Group	Mean ± SE	Levene’s statistic	Levene’s P-value)	Ddof1	Ddof2	F	P-value
Viral shedding	INa	1.24 ± 0.26	36.218	< 0.0001§	2	80.925	17.734	< 0.0001*
	IVa	0.41 ± 0.14						
	Control	2.65 ± 0.37						
Neutralizing titer	INa	80.25 ± 16.97	37.589	< 0.0001^§^	2	54.922	7.211	0.00166*
	IVa	13.94 ± 4.21						
	Control	14.5 ± 5.74						
IgG Titer	INa	1.23 ± 0.09	1.657	0.198	2		12.35	< 0.0001*
	IVa	0.73 ± 0.07						
	Control	0.73 ± 0.08						
Temperature	INa	38.59 ± 0.06	21.309	< 0.0001^§^	2	106.91	3.825	0.0249*
	IVa	38.76 ± 0.03						
	Control	38.63 ± 0.08						
Clinical score	INa	0.65 ± 0.07	162.932	< 0.0001^§^	2	91.17	15.961	< 0.0001*
	IVa	0.81 ± 0.07						
	Control	3.52 ± 0.51						

§ When Levene’s test indicated a significant difference in variance across groups, the Welch’s ANOVA replaced the standard two-way ANOVA, and Games-Howell adjustments were implemented for pairwise comparisons in place of Turkey’s HSD. *Significant difference at alpha level of ≤0.05.

Clinical examination of the control group revealed marked vaginal hyperemia, vulvar oedema and ulceration, and a fibrinous vaginal discharge with blood spots, often associated with pain during swabbing. In contrast, vaccinated goats showed no clinical signs of disease (**Figures 2B–D**). Altogether, these findings indicate that goats immunized with BoHV-4-A-gD(cp)gD(106)ΔTK were protected against the virulent CpHV-1 challenge.

### 2.3 BoHV-4-A-gD(cp)gD(106)ΔTK vaccination reduces CpHV-1 shedding from infected goats

**Figure 3:**
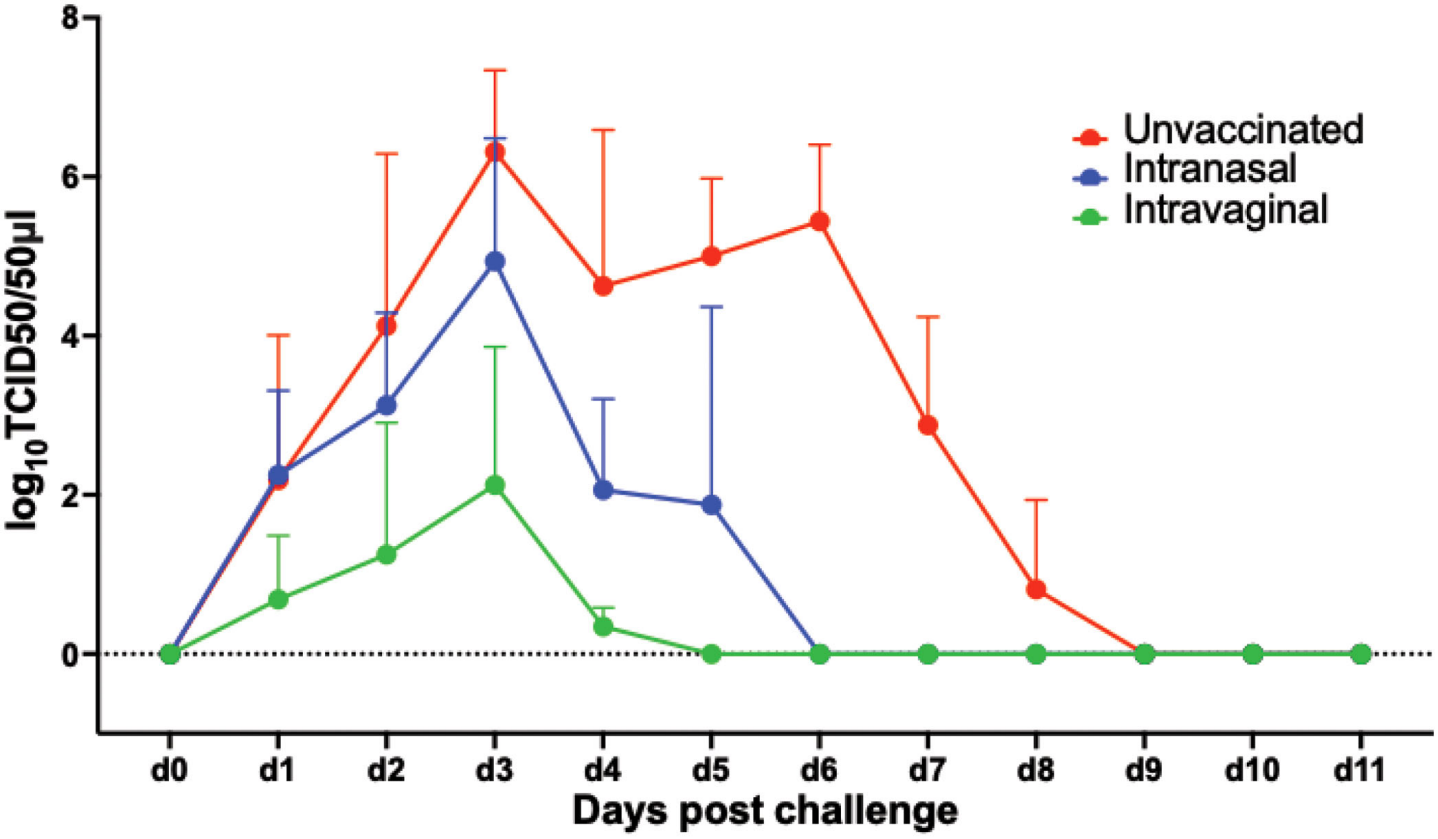
Distal and local mucosal immunization reduce CpHV-1 vaginal shedding: Mean titers of CpHV-1 in vaginal swabs obtained from the three groups of goats (unvaccinated, intranasal, and intravaginal) prior to challenge with BA-1 CpHV-1 strain (d0) up till 11 days post challenge (d11). Titres are expressed as log_10_ TCID_50_ per 50 µL of suspension derived from vaginal swabs. Vaccinated groups showed both lower levels and shorter duration of viral shedding compared to unvaccinated controls. Data were analyzed with two-way ANOVA or Welch's ANOVA followed by pairwise Tukey HSD or Games-Howell posthoc comparison, respectively.

CpHV-1 transmission is due mainly to the secretions of the infected animals in contact with healthy ones. Thus, the vaginal swabs were tested after challenge for the presence of CpHV-1. All control animals showed CpHV-1-positive swabs between days 1 and 9 post-challenge (**Figure 3**). The swabs collected from vaccinated animals, although positive for CpHV-1, intensity and duration were statistically lower, mainly for IVa vaccinated goats (**Table 2**). Thus, the BoHV-4-A-gD(cp)gD(106)ΔTK vaccine candidate impaired the shedding of virus from vaccinated animals after challenge.

**Table 2:** Pairwise *post-hoc* comparison (Games-Howell test) following Welch’s ANOVA of all analyzed variables.

Variable	Group	Comparison	Mean Diff	SE	*P-value*
Viral shedding	INa	Control	1.40	0.45	0.00758*
	IVa	Control	2.23	0.39	<0.0001*
	INa	IVa	0.83	0.30	0.0187*
IgG titer	INa	Control	0.50		0.0003*
	IVa	Control	0.01		0.998
	INa	IVa	-0.49		0.0001*
Temperature	INa	Control	0.043	0.096	0.894
	IVa	Control	-0.128	0.085	0.293
	INa	IVa	-0.172	0.066	0.029*
Neutralizing titer	INa	Control	-65.75	17.92	0.002*
	IVa	Control	-0.56	7.12	0.997
	INa	IVa	66.31	17.49	0.0016*
Clinical score	INa	Control	2.87	0.52	< 0.0001*
	IVa	Control	2.71	0.52	< 0.0001*
	INa	IVa	-0.15	0.09	0.241

*Significant difference at alpha level of ≤0.05.

### 2.4 Administration of the BoHV-4-A-gD(cp)gD(106)ΔTK vaccine triggers the development of systemic CpHV-1–specific antibodies, including functional neutralizing antibodies

**Figure 4:**
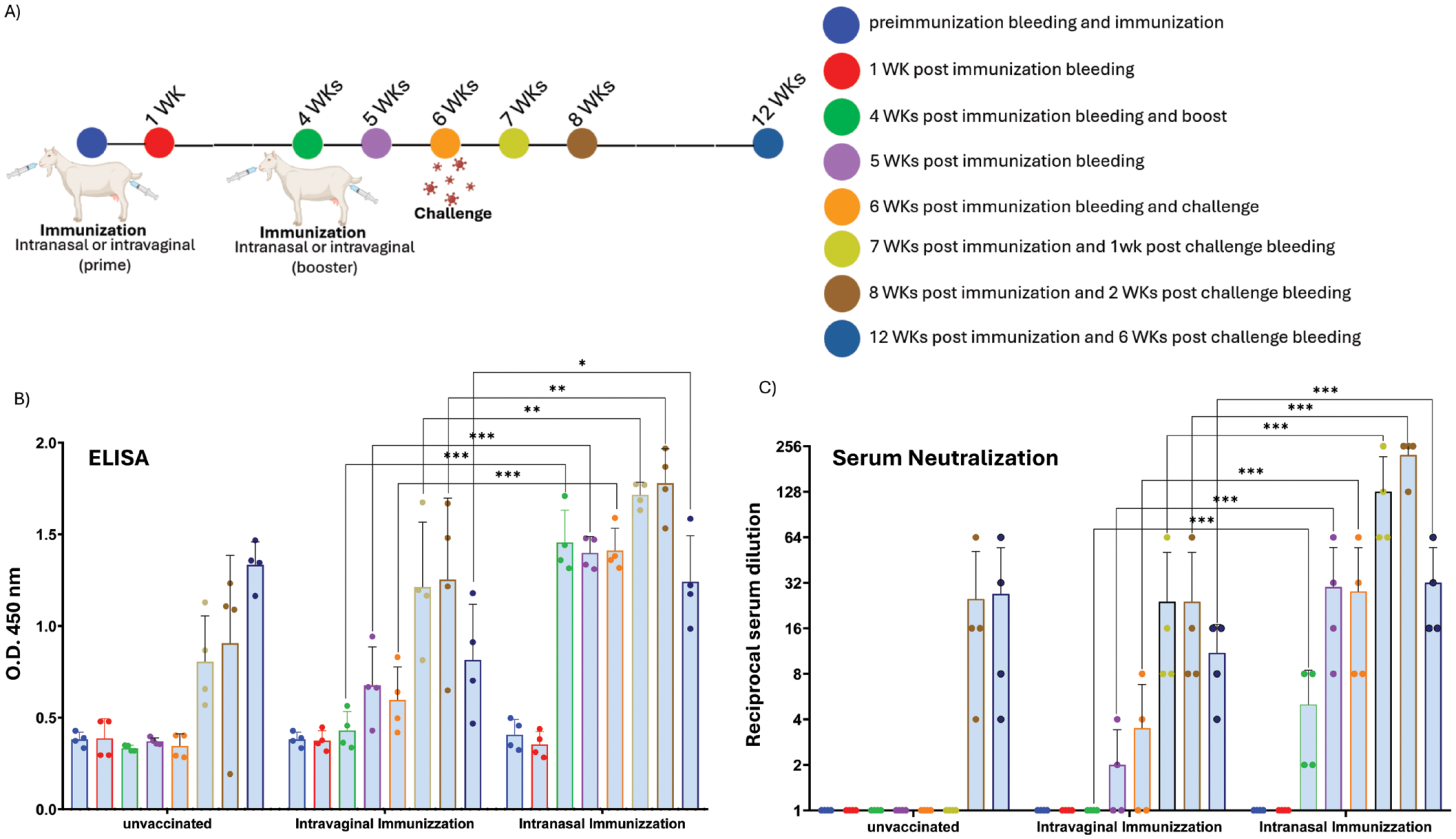
Distal and local mucosal immunization induces systemic humoral response. **(A)** Total anti-CpHV-1 gD IgG quantification through indirect ELISA of serum sample from the three groups of goats (unvaccinated, intranasal, intravaginal) expressed as O.D. 450nm values. Blood samples were collected before the first immunization (blue dot; prime), one week after the prime (red dot; 1 WK), at week 4 after the prime and prior to the booster (green dot; 4 WKs), 5 weeks after the prime (purple dot; 5 WKs), and 6 weeks after the prime and before intravaginal challenge with the BA-1 CpHV-1 strain (orange dot: 6 WKs). Additional samples were taken 7 (chartreuse dot; 7 WKs), 8 (brown dot; 8 WKs), and 12 weeks (sarin dot; 12 WKs) after the prime. **(B)** Neutralizing antibody titres of goats in the three groups obtained by serum neutralization tests expressed as a reciprocal of the serum dilution. Data were analyzed with two-way ANOVA or Welch's ANOVA followed by pairwise Tukey HSD or Games-Howell posthoc comparison, respectively. *<0.01; **<0.001; ****<0.0001

CpHV-1–specific antibodies were measured by ELISA in the sera of goats vaccinated with BoHV-4-A-gD(cp)gD(106)ΔTK. Anti–CpHV-1 gD IgG responses became detectable in INa-vaccinated animals by 4 weeks after the initial immunization, whereas IVa-vaccinated animals developed detectable titers by week 5, following the booster administered 4 weeks after the first dose. Although the booster increased antibody levels in IVa-immunized goats, INa-vaccinated animals already exhibited high titers before the booster, which then remained relatively stable throughout the study period. A slight increase in antibody levels was observed in all groups after viral challenge (**Figure 4A**). Neutralization assays were performed to assess CpHV-1–specific neutralizing antibodies in the sera of vaccinated animals. As expected, neutralizing antibodies were detected in both vaccinated groups; however, titers were significantly higher in the BoHV-4-A-gD(cp)gD(106)ΔTK INa-vaccinated group compared with the IVa-vaccinated group (**Figure 4B**). Because the temporal profile of serum neutralizing antibodies paralleled the ELISA kinetics, the overall data support the conclusion that INa immunization induced a more robust antibody immune response.

## 3 Discussion

Our results demonstrate that both local (vaginal) and distal (nasal) mucosal immunization strategies provide complete protection against CpHV-1–induced pathology in the female reproductive tract. Although the upper respiratory tract is a well-established site for vaccine delivery, the vaginal route remains comparatively underexplored and less well characterized.

In mammals, the vagina is a fibromuscular canal running from the vulva to the cervix, thereby linking the external environment with the uterus. Its mucosa is composed of a non-keratinized stratified squamous epithelium whose apical layer is permeable to both bacteria and viruses. Beneath this barrier, innate and adaptive immune cells and soluble mediators contribute to tissue capability of both pathogen susceptibility and immune defense. Lymphatic drainage primarily targets the iliac and inguinal lymph nodes. Collectively, these anatomical and immunological features make the vagina a suitable inductive site for both cellular and humoral immune responses and an attractive mucosal route for vaccination against sexually transmitted pathogens (1, 30).

To translate preclinical observations into meaningful clinical insight, an appropriate animal model is essential. While mice and guinea pigs are commonly used to study human infectious diseases (31, 32), they are suboptimal for sexually transmitted infections, as vaccine performance in these small models often correlates poorly with outcomes in larger animals. In contrast, large animal models tend to predict human vaccine efficacy more reliably (33). In this context, Caprine Herpesvirus 1 (CpHV-1) infection in goats represents a biologically relevant model due to its strong parallels with human Herpes Simplex Virus 2 (HSV-2). Both viruses show preferential tropism for the genital tract, induce vesicular–ulcerative lesions, and establish latency in the sacral ganglia (28, 34). CpHV-1 infection in goats also offers practical experimental advantages: i) infection can be consistently reproduced, generating clear and quantifiable lesions; ii) clinical scoring is straightforward; and iii) lesion severity is easy to measure (34). Moreover, goats are easy to manage, require low-cost housing, respect to cows, and allow simple blood sampling and tissue collection. These attributes make goats an excellent model for assessing recombinant vaccine vectors expressing immunodominant glycoproteins derived from different pathogens, enabling comprehensive evaluation of both immunogenicity and protective efficacy.

Demonstrating vaccine-induced immunity in this system is a key step toward identifying potential correlates of protection and supporting the rational development of HSV-2 and other reproductive tract vaccines (35). Although BoHV-4 vectors have been successfully tested in goats and sheep (28, 36), little is known about their mucosal delivery. We therefore evaluated intravaginal and intranasal administration. Ex vivo imaging showed efficient transduction of nasal mucosa but limited vaginal transduction, confined to the ectocervix, likely due to the distinct epithelial structures of the female reproductive tract. The upper tract consists of columnar epithelium with tight junctions, whereas the lower tract has stratified squamous epithelium, allowing greater permeability, particularly in the cervical transition zone (37).

Despite limited vaginal transduction, both routes supported progression to *in vivo* studies. Goats were vaccinated intravaginally (IVa) or intranasally (INa) using a prime-boost regimen and later challenged with CpHV-1. Vaccinated animals maintained normal temperatures and showed no clinical signs, while controls developed fever and genital disease. Protection was comparable between routes, indicating that both local and distal mucosal immunization can confer genital protection (38).

Notably, IVa vaccination reduced viral shedding more effectively than INa, despite lower systemic antibody responses, suggesting that local mucosal immunity plays a critical role in controlling infection. Although mechanisms were not explored due to limited reagents, this finding highlights the importance of mucosal immune responses and warrants further investigation.

In the present study, serum neutralizing antibodies emerged as a potential correlate of protection against CpHV-1 challenge. Their kinetics closely paralleled those of binding antibodies measured by ELISA, and higher titers could be associated with complete prevention of genital pathology. Although mucosal pathogens often require local immunity for optimal control, circulating neutralizing antibodies have repeatedly been shown to contribute substantially to early viral containment in alphaherpesvirus infections (39, 40). For HSV-2, elevated serum neutralizing titers correlate with reduced lesion severity, decreased viral shedding, and improved clinical outcomes in both humans and animal models (39, 40). The CpHV-1–goat system recapitulates this biology: neutralizing antibodies likely limit initial viral replication at the mucosal entry site, thereby diminishing viral dissemination and reducing the burden on local immune mechanisms. Our findings therefore support the inclusion of serum neutralizing antibody responses as a potential practical and informative immunological readout for evaluating BoHV-4–based mucosal vaccine candidates. While not the sole mediator of protection, neutralizing antibodies represent a quantifiable and biologically relevant surrogate marker that could guide dose selection, predict protective thresholds, and accelerate translation of this platform toward vaccines targeting HSV-2 and other infections of the female reproductive tract.

Instead of dissecting the precise cellular and molecular pathways involved, we concentrated on demonstrating the overall protective efficacy of the vaccination approach. Nevertheless, the observed outcomes raise several intriguing questions. For example, it remains unclear which immune compartments predominantly contribute to protection, how innate and adaptive responses might interact in this species, and whether unconventional or previously overlooked pathways could be at play. The development of suitable reagents and analytical tools would enable a deeper exploration of these possibilities, potentially revealing novel immunological features or mechanisms specific to this host. Thus, while our current findings emphasize functional protection, they also point toward several exciting avenues for mechanistic studies that could significantly expand our understanding of the immune response in this model. Extensive literature on mucosal herpesvirus immunity indicates that intravaginal immunization dynamically engages the female reproductive tract (FRT) mucosal network, which triggers targeted antigen uptake within the vaginal lamina propria, driving the local priming and retention of tissue-resident memory T cells (TRM) as well as establishing a rapid, front-line antiviral defense at the epithelial barriers (41, 42). Subsequently, the genital tract TRM cells orchestrate a highly localized antiviral microenvironment upon viral re-exposure driven by rapid antiviral cytokine release and coordinated chemokine induction (43). This mechanism is further supported by HSV-2 models, where “TRM-driven immune lockdown” is essential for suppressing viral replication and limiting shedding prior to intervention by systemic effectors. Although not directly assessed here, validating this proposed TRM seeding and retention framework and mapping its local cytokine and chemokine profile is a priority for future investigation. (44–46). In contrast, while intranasal vaccination elicited robust systemic neutralizing antibody responses, it likely generated weaker genital-specific mucosal immunity. Distal immunization sites generally provide suboptimal cues for TRM formation within the FRT, and circulating IgG—although protective—is often insufficient to fully control viral replication at mucosal entry points without the contribution of local antibodies or resident lymphocytes. Intravaginal vaccination may also promote production of secretory IgA and facilitate FcRn-mediated retention of polymeric IgG in the mucosa, enabling neutralization at the epithelial surface—compartments that are less efficiently targeted by serum-derived antibodies alone (47, 48).

Taken together, these mechanistic considerations—although not experimentally explored in the present investigation—suggest that the superior control of CpHV-1 shedding following intravaginal immunization likely reflects a more potent combination of TRM-mediated immunity, chemokine-driven effector recruitment, and tissue-proximal antibody responses. This interpretation reinforces the broader concept that local mucosal immunity is a key determinant of viral control and highlights the value of vaccine strategies capable of programming immune responses directly within the FRT microenvironment.

## 4 Materials and methods

### 4.1 Organotypic culture establishment, transduction and imaging

Complete reproductive tracts and rostral portions of the head, including the nasal cavities, were collected from two 1-year-old female goats at a commercial abattoir and transported to the laboratory on ice. Vaginal organotypic cultures were established as previously described with minor modifications (49). Briefly, after removal of surrounding connective tissue, full-thickness sections comprising the ectocervix and the cranial third of the vagina were dissected, rinsed in PBS with 50 mg/mL gentamicin (Gibco), and placed in a 6-well plate with the ectocervix oriented upwards. The nasal cavities were opened by dissecting the rostrum along the median line and removing the nasal septa to expose the turbinates. Turbinates samples were then dissected rinsed in PBS and placed into a 6-well plate. Wells were then filled with 3 mL of cEMEM with 10% FBS containing 3 x 10^7^ TCID_50_ of BoHV-4-A-Luc ΔTK or parental BoHV4 as a control. Organotypic cultures were incubated at 37 °C/5% CO2. Twenty-four hours after transduction, the culture medium was removed and replaced with fresh cEMEM supplemented with 10% D-luciferin (Perkin Elmer, Milan, MI, Italy; 1 mg/mL in 0.9% sodium chloride), gently applied to the mucosal surface. Bioluminescent imaging of luciferase activity was then carried out using a CCD camera system (ChemiDoc XRS+, BioRad, Segrate, MI, Italy). Plates containing BoHV-4-A-LucΔTK–transduced organotypic cultures, along with control cultures, were imaged using standard chemiluminescence detection settings in signal accumulation mode. Images were subsequently visualized in both grayscale and pseudocolor formats.

### 4.2 Transduction intensity quantification

Transduction efficiency was assessed in vaginal, cervical, and nasal mucosae after exposure to BoHV-4-A-LucΔTK or BoHV4 using previously described quantification methods (49). In brief, image quantification was implemented using a custom Python workflow integrating essential libraries for high-throughput processing and visualization. Chemiluminescence images of BoHV-4-A-LucΔTK- or BoHV4-exposed specimens were binarized into 2D arrays using an inverse threshold technique with a fixed intensity cutoff of 32. Following binarization, pixel intensity was mapped and assigned values of 0 or 255 relative to the cutoff. Contour boundaries of each segment were extracted and applied Simple Chain Approximation algorithm to compress the boundary points while preserving the geometric structure. Transduction intensity was quantified by calculating the cumulative area of all identified contours, expressed in square pixels (*psq*) units (**Supplementary Figure 1**) (49).

### 4.3 Cells

HEK 293T (human embryo kidney cells; ATCC: CRL-11268) and MDBK (Mardin Darby Bovine Kidney; ATCC: CRL-6071) were cultured in complete Eagle’s minimal essential medium (cEMEM) supplemented with 2 mM of L-glutamine, 1 mM of sodium pyruvate, 100 IU/mL of penicillin, 100 µg/mL of L-glutamine, 0.25 µg/mL of amphotericin B, and 10% FBS. Cells were cultured in a humified incubator at 37^0^C/5% CO_2_. All supplements for the culture medium were purchased from Gibco (Segrate, MI, Italy).

### 4.4 Viruses

The generation, characterization amplification procedure of BoHV-4-A-gD(cp)gD(106)ΔTK and BoHV-4-A-Luc ΔTK has been fully described in previous studies (28, 29). BA-1 strain of CpHV-1, isolated from a latently infected goat, was cultured and titrated in MDBK cells. The viral stocks containing 10^7^ 50% tissue culture infectious doses (TCID_50_)/50 µL were stored at -80 °C and used for the experiments. For virus titration, the stock virus was serially 10-fold diluted and inoculated in quadruplicate onto MDBK cells in 96-well microtiter plates, incubated at 37 °C and maintained in a 5% CO_2_ atmosphere. The titer was read after 3 days of incubation by observation of cytopathic effect (CPE).

### 4.5 Construct generation and Sec-gD protein production

The generation of pCMV-Sec-gD construct, delivering CpHV-1 gD secreted fragment (Sec-gD) was previously described (50). Sec-gD was produced in HEK 293T cell, transiently transfected with pCMV-Sec-gD. The protocol has been fully described (50). Briefly, HEK 293T cells were plated into 175 cm^2^ flasks (5 × 10^6^ cells/flask) and grown till sub-confluency, then the culture medium was removed and replaced with the transfection mixture. pCMV-Sec-gD or pEGFP-C1 (as a mock control; Clontech, San Jose, CA, USA) were transfected into the cells using polyethylenimine (PEI) transfection reagent (Polysciences, Inc., Warrington, PA, USA). The DNA was mixed with PEI in a ratio of 1:2.5 (DNA: PEI) in 3.500 mL of serum-free Dulbecco’s modified essential medium (DMEM) with high glucose (Euroclone, Pero, Italy) and incubated for 15 min at room temperature. Next, 4× volumes of serum-free medium were added, and the transfection solution was transferred onto the cells monolayer and left for 6 h at 37 °C with 5% CO_2_, in a humidified incubator. The transfection mixture was then replaced with 21 mL of DMEM/F12 (Ham’s F12 Nutrient Mixture; Euroclone Pero, Italy) (1:1) and incubated for 48 h. The cell supernatants, containing Sec-gD protein, were then harvested, clarified at 2,500 rpm at 4 °C and stored at −80 °C.

### 4.6 Animals and experimental infection

Experimental protocols for goat infection were duly authorized (code 48E68, min aut. 869/15.11.2021) and conducted at the authorized University of Bari experimental animal facility (authorization no. 06/2023-UT).

Twelve 2-3-year-old Maltese and Saanen crossbred female goats without neutralizing antibodies to CpHV-1were used in this study. Prior to experimentation, the goats were held under controlled environmental conditions and examined daily for clinical evidence of disease. At the start of the trial, the goats were randomly divided into three groups. Goats in group A (A1, A2, A3, and A4) were vaccinated intranasally (INa) while the goats in group B (B1, B2, B3, and B4) were vaccinated intravaginally (IVa). Each goat in the vaccinated groups (A and B) received 1 mL of BoHV-4-A-gD(cp)gD(106)ΔTK at a dose of 10^6^ TCID_50_/mL. Four unvaccinated goats were kept as controls and received, both INa and IVa, 1 ml of medium. After 28 days, goats were revaccinated with a booster dose of BoHV-4-A-gD(cp)gD(106)ΔTK via intranasal (group A) and intravaginal (group B) routes respectively. Two weeks post second (booster) vaccination, all goats in the vaccinated (A and B) and control groups were challenged intravaginally with 3 mL of BA-1 CpHV-1 strain (3.56 × 10^6^ TCID50/mL). All animals were clinically examined, and rectal temperatures were measured daily for 11 days post CpHV-1 challenge (**Supplementary Table 1**). Clinical signs of hyperemia, edema, vesicular lesions and pain were graded as: 0 (absent), 1 (mild), 2 (moderate), and 3 (severe). Temperature elevations above normal (38.5 °C) were graded as 1 (>0.5 °C to 1 °C), 2 (1.1 °C–1.5 °C), and 3 (>1.5 °C). The total daily clinical score for each animal was recorded from day 0 (pre-challenge) to day 11 post-challenge. During the period, vaginal swab samples were collected daily for virus excretion titre determination while blood samples were collected at Days 0, 1, 4, 5, 6, 7, 8, and 14 for serology. Antibody titre determination was conducted on all goats as illustrated in **Figure 2A**: before the first immunization (blue dot; prime), one week after the prime (red dot; 1 WK), at week 4 after the prime and prior to the booster (green dot; 4 WKs), 5 weeks after the prime (purple dot; 5 WKs), and 6 weeks after the prime and before intravaginal challenge with the BA-1 CpHV-1 strain (orange dot: 6 WKs). Additional samples were taken 7 (chartreuse dot; 7 WKs), 8 (brown dot; 8 WKs), and 12 weeks (sarin dot; 12 WKs) after the prime.

### 4.7 Serum neutralization assay

Goat sera were obtained by venipuncture in EDTA-free vacutainer and heat inactivated at 56 °C for 30 min. Subsequently, serial two-fold dilutions of each serum from 1:2 up to 1:256 were mixed with 100 TCID50 of the BA-1 strain of CpHV-1 in 96-well microtiter plates to perform Serum Neutralization Assay. The plates were incubated for 90 min at room temperature, and then, 10^4^ MDBK cells in a volume of 100 µL of cEMEM were added to each well, and the plates were then incubated for 3 days at 37 °C in a 5% CO2 humidified environment. Readings were made when CPEs were complete in the virus control cultures, and the titer of each serum was expressed as the reciprocal of the highest dilution neutralizing the virus in the well.

### 4.8 ELISA procedure

ELISA on goat serum sample was performed as previously described (50). Briefly, 50 ng/well of Sec-gD protein supernatant, diluted in 0.1 M carbonate/bicarbonate buffer at pH 9.6, were used to coat the ninety-six-well microplates (Microlon High Binding, Greiner Bio-One, Kremsmünster, Austria), overnight at 4 °C. Next, 1% Bovine Serum Albumin (BSA) in Phosphate Buffer saline (PBS) (Sigma Aldrich by Merck, Rome, Italy) blocking step was performed and goat serum samples were then added and incubated for 1 h at room temperature at different twofold dilutions (1/10, 1/20, 1/40, 1/80). Serum samples were diluted in exhausted DMEM/F12 without serum, collected from HEK 293T, grown for 48 h. After three washing steps in PBS, 50 µL of donkey anti-goat IgG-HRP (Santa Cruz Biotechnology, Heidelberg, Germany) diluted 1:5,000 was added to each well, and the plate was incubated as above. Following the final washing step, the reaction was developed with 3,3′,5,5′-tetramethylbenzidine (Merck, Rome, Italy), stopped with 0.2 M H_2_SO_4_ and read at 450 nm.

### 4.9 Virus excretion titre

Vaginal swabs were immersed in DMEM and centrifuges at 8000rpm for 3 min. The supernatant was then treated with 10% mixture of antibiotics (5000 UI/mL penicillin, 2500 µg/mL streptomycin, 10 µg/mL amphotericin) for 30 min at room temperature and inoculated onto monolayers of MDBK cells and titrated as described above.

### 4.10 Statistical analysis

All analysis was implemented using Python version 3.9.18 (https://docs.python.org/3/reference/index.html) with the initiation of core libraries and their dependencies in the Python programming environment. The statsmodels library with its Ordinary Least Squares (OLS) functionality was utilized for model estimation. A Two-Way Analysis of Variance (ANOVA) was conducted to compare the mean virus titres measured as log10 tissue culture infectious doses (TCID_50_)/50 µL, mean temperature across the various experimental goat groups, mean clinical scores and antibodies titers. A calculated p-value of 0.05 or less, together with its corresponding F-statistic, was considered statistically significant.

Levene’s test was performed to evaluate homogeneity of variance across groups. Where equal variance was violated, Welch’s ANOVA was computed to mitigate the loss of statistical power and the inflated Type I error rates associated with standard two-way ANOVA. When significant effect was observed, the dataset was further evaluated using Games-Howell *post-hoc* pairwise comparisons utilizing degrees-of-freedom adjustments and a Studentized range distribution to control the family-wise error rate. Conversely, when the assumption of variance was met, a standard two-way ANOVA was conducted, followed by Turkey’s HSD pairwise comparisons to identify significant mean differences. The result was further validated with the Dunnett’s Test performed with fewer comparisons which offers the advantage of increased statistical power. The results were presented in result’s table and figures. Data visualization and statistical rendering were performed using GraphPad Prism (Version 8.0.1, GraphPad Software).

## Data Availability

The raw data supporting the conclusions of this article will be made available by the authors, without undue reservation.
