# Supplementary material for: Distal and local mucosal immunization with a BoHV-4-based vector delivering CpHV-1 gD confers protection against intravaginal CpHV-1 challenge in goats
Source: Front Immunol. 2026 Jul 14;17:1884557. doi: 10.3389/fimmu.2026.1884557 (PMC13407295; doi:10.3389/fimmu.2026.1884557)
Supplement: Supplementary file 1 [file DataSheet1.pdf]

Vaginal_Transduction_Quantification_v0.6_23.04.2026

April 23, 2026

All intellectual property rights associated with this code are exclusively held by authors, who developed and implemented it for the purposes of this study.

0.0.1 Code Implementation

The following libraries and its respective dependencies were imported into the Python programming environment: 1. Numerical Python (NumPy) as np for array operations 2. Matplotlib and Seaborn for data visualization 3. Pandas for data analysis 4. Open-Source Computer Vision (cv2) for image processing

[1]:
```
# import all libraries
import numpy as np
import matplotlib.pyplot as plt
import seaborn as sns
import pandas as pd
import cv2
```

Create Image Path

[2]:
```
img_path1 = r"C:\Users\Utente\Downloads\vaginal_transduction_img\img_1a.png"
img_path2 = r"C:\Users\Utente\Downloads\vaginal_transduction_img\img_2a.png"
img_path3 = r"C:\Users\Utente\Downloads\vaginal_transduction_img\img_3a.png"
img_path4 = r"C:\Users\Utente\Downloads\vaginal_transduction_img\img_4a.png"
```

Read and resize image

[3]:
```
# Specify the desired width and height
desired_width = 240
desired_height = 235

# Read first image set
img1 = cv2.imread(img_path1)
img1 = cv2.resize(img1, (desired_width, desired_height))
img2 = cv2.imread(img_path2)
img2 = cv2.resize(img2, (desired_width, desired_height))
img3 = cv2.imread(img_path3)
img3 = cv2.resize(img3, (desired_width, desired_height))
img4 = cv2.imread(img_path4)
img4 = cv2.resize(img4, (desired_width, desired_height))
```

Inspect images

[4]:
```
fig, axes = plt.subplots(ncols=4, figsize=(7,3))
# first row
axes[0].imshow(cv2.cvtColor(img1, cv2.COLOR_BGR2RGB))
axes[0].text(0.95, 1.05, fontsize=6, transform=axes[0].transAxes, va='bottom', ha='right', s='cervix + BoHV-4-A-LucΔTK')

axes[1].imshow(cv2.cvtColor(img2, cv2.COLOR_BGR2RGB))
axes[1].text(0.75, 1.05, fontsize=6, transform=axes[1].transAxes, va='bottom', ha='right', s='cervix + BoHV-4')

axes[2].imshow(cv2.cvtColor(img3, cv2.COLOR_BGR2RGB))
axes[2].text(0.80, 1.05, fontsize=6, transform=axes[2].transAxes, va='bottom', ha='right', s='nasal turbinate \n+ BoHV-4-A-LucΔTK')

axes[3].imshow(cv2.cvtColor(img4, cv2.COLOR_BGR2RGB))
axes[3].text(0.70, 1.05, fontsize=6, transform=axes[3].transAxes, va='bottom', ha='right', s='nasal turbinate\n+ BoHV-4')

for ax in axes.flat:
    ax.axis('off')

plt.show()
```

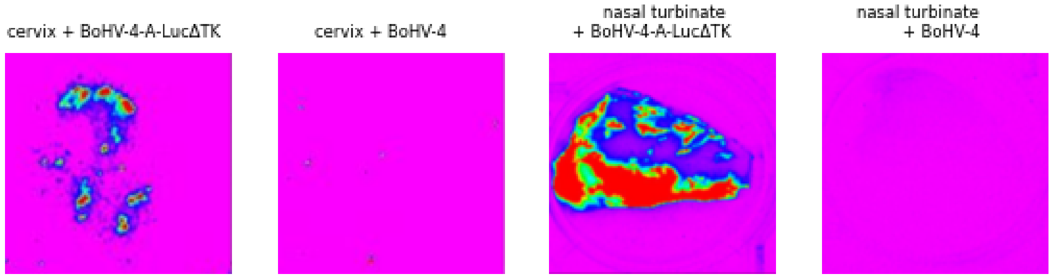

Convert to HSV

[5]:
```
hsv1 = cv2.cvtColor(img1, cv2.COLOR_BGR2HSV)
hsv2 = cv2.cvtColor(img2, cv2.COLOR_BGR2HSV)
hsv3 = cv2.cvtColor(img3, cv2.COLOR_BGR2HSV)
hsv4 = cv2.cvtColor(img4, cv2.COLOR_BGR2HSV)
```

The colour values of the HSV-transformed images are adjusted for fine-grained control by defining an upper and lower colour range boundaries for green and blue hues respectively with a threshold cutoff 37 using the threshold-binary-inverse method.

[6]:
```
threshold_value = 37
max_value = 255

# Define the lower and upper bounds for blue color in HSV (Adjust values as␣
↪needed)
lower_blue = np.array([71, 71, 71])
upper_blue = np.array([255, 255, 255])

lower_green = np.array([71, 71, 71])
upper_green = np.array([255, 255, 255])

_, background1 = cv2.threshold(cv2.inRange(hsv1, lower_blue, upper_blue),␣
↪threshold_value, max_value, cv2.THRESH_BINARY_INV)
_, background2 = cv2.threshold(cv2.inRange(hsv2, lower_blue, upper_blue),␣
↪threshold_value, max_value, cv2.THRESH_BINARY_INV)
_, background3 = cv2.threshold(cv2.inRange(hsv3, lower_green, upper_green),␣
↪threshold_value, max_value, cv2.THRESH_BINARY_INV)
_, background4 = cv2.threshold(cv2.inRange(hsv4, lower_blue, upper_blue),␣
↪threshold_value, max_value, cv2.THRESH_BINARY_INV)
```

[7]:
```
fig, axes = plt.subplots(ncols=4, figsize=(7,3))
# first row
axes[0].imshow(background1)
axes[0].text(0.95, 1.05, fontsize=6, transform=axes[0].transAxes, va='bottom',␣
↪ha='right', s='cervix + BoHV-4-A-LucΔTK')

axes[1].imshow(background2)
axes[1].text(0.75, 1.05, fontsize=6, transform=axes[1].transAxes, va='bottom',␣
↪ha='right', s='cervix + BoHV-4')

axes[2].imshow(background3)
axes[2].text(0.80, 1.05, fontsize=6, transform=axes[2].transAxes, va='bottom',␣
↪ha='right', s='nasal turbinate \n+ BoHV-4-A-LucΔTK')

axes[3].imshow(background4)
axes[3].text(0.70, 1.05, fontsize=6, transform=axes[3].transAxes, va='bottom',␣
↪ha='right', s='nasal turbinate\n+ BoHV-4')

for ax in axes.flat:
    ax.axis('off')

plt.show()
```

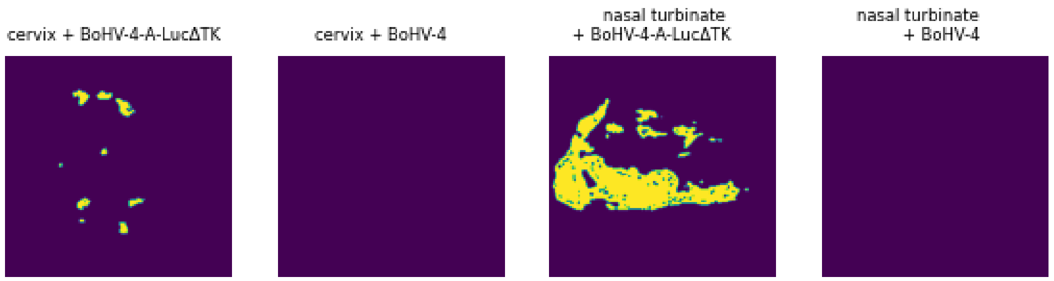

Compute the respective contours

[8]:
```
contours1, _ = cv2.findContours(background1, cv2.RETR_EXTERNAL, cv2.
  ↪CHAIN_APPROX_SIMPLE)
contours2, _ = cv2.findContours(background2, cv2.RETR_EXTERNAL, cv2.
  ↪CHAIN_APPROX_SIMPLE)
contours3, _ = cv2.findContours(background3, cv2.RETR_EXTERNAL, cv2.
  ↪CHAIN_APPROX_SIMPLE)
contours4, _ = cv2.findContours(background4, cv2.RETR_EXTERNAL, cv2.
  ↪CHAIN_APPROX_SIMPLE)
```

The combined area of all estimated contours is calculated in pixel squared unit - a measure of the bioluminescence intensity.

[9]:
```
all_contours = [
    contours1, contours2, contours3, contours4
]

areas = [sum(cv2.contourArea(cnt) for cnt in group) for group in all_contours]
print(areas)
```

```
[637.5, 0, 8116.0, 0]
```

[10]:
```
cervix_qnt,vagina_qnt,nasal_qnt,vulva_qnt = areas
```

Visualize the data

[11]:
```
df = {
    "image": ['cervix + BoHV-4-A-LucΔTK',
              'cervix + BoHV-4',
              'nasal turbinate + BoHV-4-A-LucΔTK',
              'nasal turbinate + BoHV-4'],
    "biolum_quant": [cervix_qnt,vagina_qnt,nasal_qnt,vulva_qnt],
    "category":['reproductive','reproductive','respiratory','respiratory']
    }
df = pd.DataFrame(df)
df.set_index(df.columns[0])
```

[11]:

```
                                 biolum_quant      category
image
cervix + BoHV-4-A-LucΔTK                637.5  reproductive
cervix + BoHV-4                           0.0  reproductive
nasal turbinate + BoHV-4-A-LucΔTK      8116.0   respiratory
nasal turbinate + BoHV-4                  0.0   respiratory
```

[12]:

```python
import textwrap

plt.figure(figsize=(7, 7))
bar_width = 0.6

sns.barplot(data=df, x="image", y="biolum_quant", hue='category')

plt.ylabel('Transduction Quantification (pixel squared)')
plt.title('Plot of Transduction Intensity Quantification',
         fontsize=15, fontweight='bold')
plt.grid(axis='y', linestyle='--', alpha=0.7)
plt.legend(loc='best', bbox_to_anchor=(0.77, 0., 0.65, 1.02))
plt.xlabel('')
wrapped_labels = [textwrap.fill(p, width=10) if i > 0 else p
    for i, p in enumerate(df['image'])]
plt.xticks(range(len(df['image'])), wrapped_labels, fontsize=10)

plt.show()
```

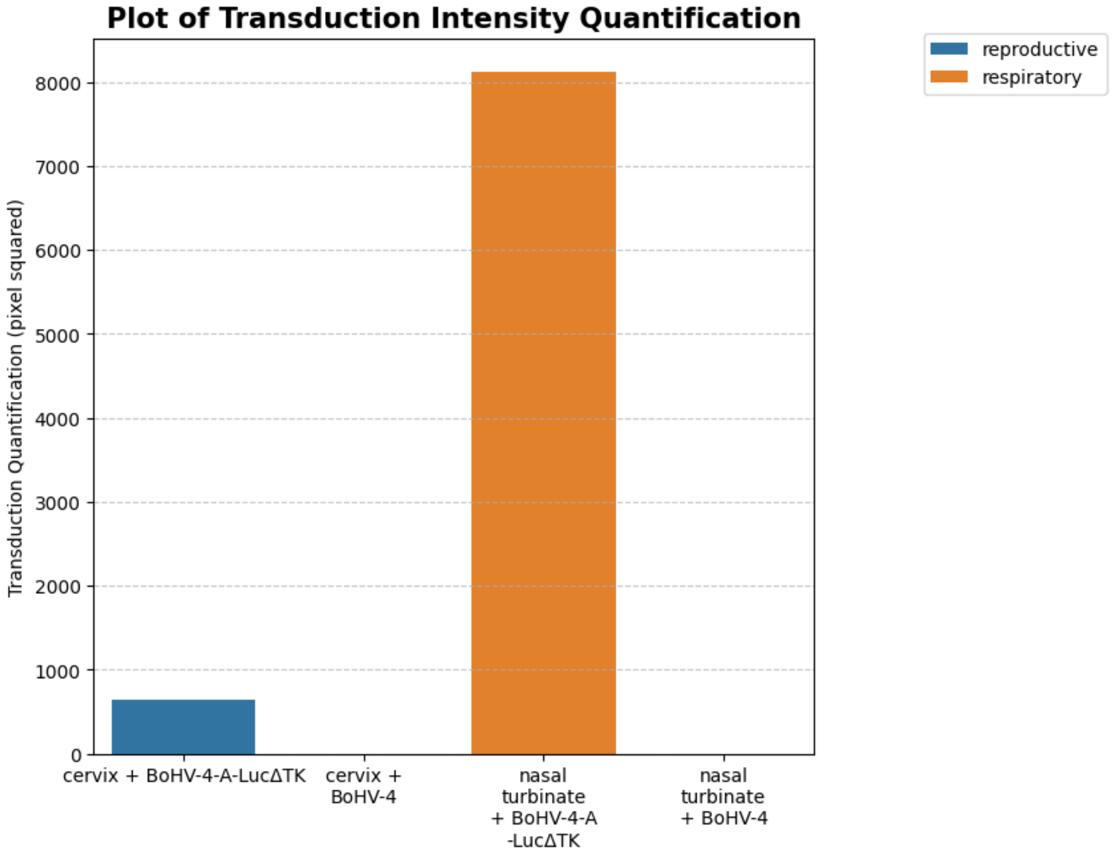
Plot of Transduction Intensity Quantification
Transduction Quantification (pixel squared)
0
1000
2000
3000
4000
5000
6000
7000
8000
cervix + BoHV-4-A-LucΔTK
cervix + BoHV-4
nasal turbinate + BoHV-4-A -LucΔTK
nasal turbinate + BoHV-4
reproductive
respiratory
