# Supplementary material for: Distal and local mucosal immunization with a BoHV-4-based vector delivering CpHV-1 gD confers protection against intravaginal CpHV-1 challenge in goats
Source: Front Immunol. 2026 Jul 14;17:1884557. doi: 10.3389/fimmu.2026.1884557 (PMC13407295; doi:10.3389/fimmu.2026.1884557)
Supplement: Supplementary file 2 [file DataSheet2.pdf]

Semi-quantitative scoring system (0 to 3) across three distinct criteria: hyperemia, edema, and lesion severity. Individual scores were summed to calculate a cumulative Total Clinical Score using the following formula: Total Score = Hyperaemia(0-3) + Edema(0-3) + Lesions(0-3)

		Days post challenge													
**Goat no.**	**Clinical signs**	**0**	**1**	**2**	**3**	**4**	**5**	**7**	**8**	**9**	**10**	**11**	**12**	**13**	**14**
A1	hyperemia	0	0	0	0	0	0	0	0	0	0	0	0	0	0
	edema	0	0	0	0	0	0	0	0	0	0	0	0	0	0
	lesion	0	0	0	0	0	0	0	0	0	0	0	0	0	0
	temperature	0	0	0	0	0	1	0	0	0	1	0	0	0	0
	**TOTAL SCORE**	**0**	**0**	**0**	**0**	**0**	**1**	**0**	**0**	**0**	**1**	**0**	**0**	**0**	**0**
A2	hyperemia	0	0	0	0	0	0	0	0	0	0	0	0	0	0
	edema	0	0	0	0	0	0	0	0	0	0	0	0	0	0
	lesion	0	0	0	0	0	0	0	0	0	0	0	0	0	0
	temperature	0	1	1	1	1	1	1	1	1	1	1	1	1	1
	**TOTAL SCORE**	**0**	**1**	**1**	**1**	**1**	**1**	**1**	**1**	**1**	**1**	**1**	**1**	**1**	**1**
A3	hyperemia	0	0	0	0	0	0	0	0	0	0	0	0	0	0
	edema	0	0	0	0	0	0	0	0	0	0	0	0	0	0
	lesion	0	0	0	0	0	0	0	0	0	0	0	0	0	0
	temperature	0	0	0	1	1	1	0	1	0	1	1	1	1	1
	**TOTAL SCORE**	**0**	**0**	**0**	**1**	**1**	**1**	**0**	**1**	**0**	**1**	**1**	**1**	**1**	**1**
A4	hyperemia	0	0	0	0	0	0	0	0	0	0	0	0	0	0
	edema	0	0	0	0	0	0	0	0	0	0	0	0	0	0
	lesion	0	0	0	0	0	0	0	0	0	0	0	0	0	0
	temperature	0	0	1	1	1	1	0	1	0	1	1	1	1	1
	**TOTAL SCORE**	**0**	**0**	**1**	**1**	**1**	**1**	**0**	**1**	**0**	**1**	**1**	**1**	**1**	**1**
B1	hyperemia	0	0	0	0	0	0	0	0	0	0	0	0	0	0
	edema	0	0	0	0	0	0	0	0	0	1	1	0	0	0
	lesion	0	0	0	0	0	0	0	0	0	0	0	0	0	0
	temperature	0	1	1	1	1	1	1	1	1	1	1	1	1	1
	**TOTAL SCORE**	**0**	**1**	**1**	**1**	**1**	**1**	**1**	**1**	**1**	**2**	**2**	**1**	**1**	**1**
B2	hyperemia	0	0	0	0	0	0	0	0	0	0	0	0	0	0
	edema	0	0	0	0	0	0	0	0	0	0	0	0	0	0
	lesion	0	0	0	0	0	0	0	0	0	0	0	0	0	0
	temperature	0	1	1	1	1	1	1	1	1	1	1	1	1	1
	**TOTAL SCORE**	**0**	**1**	**1**	**1**	**1**	**1**	**1**	**1**	**1**	**1**	**1**	**1**	**1**	**1**
B3	hyperemia	0	0	0	0	0	0	0	0	0	0	0	0	0	0
	edema	0	0	0	0	0	0	0	0	0	0	0	0	0	0
	lesion	0	0	0	0	0	0	0	0	0	0	0	0	0	0
	temperature	0	0	0	1	1	1	0	1	1	1	0	0	0	0
	**TOTAL SCORE**	**0**	**0**	**0**	**1**	**1**	**1**	**0**	**1**	**1**	**1**	**0**	**0**	**0**	**0**
B4	hyperemia	0	0	0	0	0	0	0	0	0	0	0	0	0	0
	edema	0	0	0	0	0	0	0	0	0	0	0	0	0	0
	lesion	0	0	0	0	0	0	0	0	0	0	0	0	0	0
	temperature	0	0	1	1	1	1	1	1	1	1	0	0	0	0
	**TOTAL SCORE**	**0**	**0**	**1**	**1**	**1**	**1**	**1**	**1**	**1**	**1**	**0**	**0**	**0**	**0**
C1	hyperemia	0	0	0	2	3	3	3	2	2	2	1	0	0	0
	edema	0	0	1	2	3	3	3	2	2	2	1	1	1	1
	lesion	0	0	0	2	3	3	3	2	2	2	1	0	0	0
	temperature	0	0	1	1	1	1	1	1	1	1	0	0	0	1
	**TOTAL SCORE**	**0**	**0**	**2**	**7**	**10**	**10**	**10**	**7**	**7**	**7**	**3**	**1**	**1**	**2**
C2	hyperemia	0	0	0	1	2	3	3	2	2	2	1	0	0	0
	edema	0	0	0	0	2	3	3	2	2	2	1	1	1	0
	lesion	0	0	0	0	2	3	3	2	2	2	1	0	0	0
	temperature	0	1	1	2	1	1	1	2	1	1	1	0	1	1
	**TOTAL SCORE**	**0**	**1**	**1**	**3**	**7**	**10**	**10**	**8**	**7**	**7**	**4**	**1**	**2**	**1**
C3	hyperemia	0	0	0	1	2	3	3	3	2	1	0	0	0	0
	edema	0	0	0	1	2	3	3	3	2	1	0	0	0	0
	lesion	0	0	0	1	2	3	3	3	2	1	0	0	0	0
	temperature	0	0	1	1	1	1	0	0	0	0	0	0	0	0
	**TOTAL SCORE**	**0**	**0**	**1**	**4**	**7**	**10**	**9**	**9**	**6**	**3**	**0**	**0**	**0**	**0**
C4	hyperemia	0	0	0	0	0	0	0	0	0	0	0	0	0	0
	edema	0	0	0	0	0	0	0	0	0	0	0	0	0	0
	lesion	0	0	0	0	0	0	0	0	0	0	0	0	0	0
	temperature	0	1	0	1	1	1	0	0	1	0	0	0	0	0
	**TOTAL SCORE**	**0**	**1**	**0**	**1**	**1**	**1**	**0**	**0**	**1**	**0**	**0**	**0**	**0**	**0**

Mucosal Clinical Scoring Matrix

**Score**	**Hyperemia**	**Edema**	**Lesion Severity**
0 (Normal)	Uniformly pale pink, moist, and glistening mucosa.	Sharp, defined, pliable mucosal folds; normal anatomy.	Intact, smooth epithelial surface; free of architectural defects.
1 (Mild)	Pink-to-red discoloration; capillary dilation restricted to isolated patches.	Slight puffiness/thickening of folds; tissue remains pliable; lumen entirely patent.	Superficial, focal epithelial abrasions; no deep tissue loss or exudate.
2 (Moderate)	Diffuse, bright red coloration; widespread vascular engorgement; no spontaneous hemorrhage.	Prominent thickening and blunting of folds; noticeable narrowing of the vaginal lumen.	Well-demarcated ulcers penetrating to the basement membrane; covered by fibrinopurulent exudate.
3 (Severe)	Intense, dark red to cyanotic purple discoloration; marked petechiae or ecchymotic hemorrhages.	Tense, rigid, translucent swelling; normal folds obliterated; vaginal lumen occluded.	Extensive, coalescing deep ulcers; prominent necrosis, mucosal sloughing, and heavy discharge.
